# The influence of age‐associated comorbidities on responses to combination antiretroviral therapy in older people living with HIV

**DOI:** 10.1002/jia2.25228

**Published:** 2019-02-25

**Authors:** Mi Young Ahn, Awachana Jiamsakul, Suwimon Khusuwan, Vohith Khol, Thuy T Pham, Romanee Chaiwarith, Anchalee Avihingsanon, Nagalingeswaran Kumarasamy, Wing Wei Wong, Sasisopin Kiertiburanakul, Sanjay Pujari, Kinh V Nguyen, Man Po Lee, Adeeba Kamarulzaman, Fujie Zhang, Rossana Ditangco, Tuti P Merati, Evy Yunihastuti, Oon Tek Ng, Benedict L H Sim, Junko Tanuma, Winai Ratanasuwan, Jeremy Ross, Jun Yong Choi, IeDEA Asia‐Pacific

**Affiliations:** ^1^ Department of Internal Medicine Yonsei University College of Medicine Seoul South Korea; ^2^ AIDS Research Institute Yonsei University College of Medicine Seoul South Korea; ^3^ Department of Internal Medicine Seoul Medical Center Seoul South Korea; ^4^ The Kirby Institute UNSW Sydney NSW Australia; ^5^ Chiangrai Prachanukroh Hospital Chiang Rai Thailand; ^6^ National Center for HIV/AIDS, Dermatology & STDs Phnom Penh Cambodia; ^7^ Bach Mai Hospital Hanoi Vietnam; ^8^ Research Institute for Health Sciences Chiang Mai Thailand; ^9^ HIV‐NAT/Thai Red Cross AIDS Research Centre Bangkok Thailand; ^10^ Chennai Antiviral Research and Treatment Clinical Research Site (CART CRS) YRGCARE Medical Centre VHS Chennai India; ^11^ Taipei Veterans General Hospital Taipei Taiwan; ^12^ Faculty of Medicine Ramathibodi Hospital Mahidol University Bangkok Thailand; ^13^ Institute of Infectious Diseases Pune India; ^14^ National Hospital for Tropical Diseases Hanoi Vietnam; ^15^ Queen Elizabeth Hospital Hong Kong SAR China; ^16^ University Malaya Medical Centre Kuala Lumpur Malaysia; ^17^ Beijing Ditan Hospital Capital Medical University Beijing China; ^18^ Research Institute for Tropical Medicine Muntinlupa City Philippines; ^19^ Faculty of Medicine Udayana University & Sanglah Hospital Bali Indonesia; ^20^ Faculty of Medicine Universitas Indonesia ‐ Dr. Cipto Mangunkusumo General Hospital Jakarta Indonesia; ^21^ Tan Tock Seng Hospital Singapore City Singapore; ^22^ Hospital Sungai Buloh Sungai Buloh Malaysia; ^23^ National Center for Global Health and Medicine Tokyo Japan; ^24^ Faculty of Medicine Siriraj Hospital Mahidol University Bangkok Thailand; ^25^ TREAT Asia amfAR ‐ The Foundation for AIDS Research Bangkok Thailand

**Keywords:** HIV, cART, age‐associated comorbidity, immunological failure, virological failure, TAHOD (TREAT Asia HIV Observational Database)

## Abstract

**Introduction:**

Multiple comorbidities among HIV‐positive individuals may increase the potential for polypharmacy causing drug‐to‐drug interactions and older individuals with comorbidities, particularly those with cognitive impairment, may have difficulty in adhering to complex medications. However, the effects of age‐associated comorbidities on the treatment outcomes of combination antiretroviral therapy (cART) are not well known. In this study, we investigated the effects of age‐associated comorbidities on therapeutic outcomes of cART in HIV‐positive adults in Asian countries.

**Methods:**

Patients enrolled in the TREAT Asia HIV Observational Database cohort and on cART for more than six months were analysed. Comorbidities included hypertension, diabetes, dyslipidaemia and impaired renal function. Treatment outcomes of patients **≥**50 years of age with comorbidities were compared with those <50 years and those **≥**50 years without comorbidities. We analysed 5411 patients with virological failure and 5621 with immunologic failure. Our failure outcomes were defined to be in‐line with the World Health Organization 2016 guidelines. Cox regression analysis was used to analyse time to first virological and immunological failure.

**Results:**

The incidence of virologic failure was 7.72/100 person‐years. Virological failure was less likely in patients with better adherence and higher CD4 count at cART initiation. Those acquiring HIV through intravenous drug use were more likely to have virological failure compared to those infected through heterosexual contact. On univariate analysis, patients aged <50 years without comorbidities were more likely to experience virological failure than those aged ≥50 years with comorbidities (hazard ratio 1.75, 95% confidence interval (CI) 1.31 to 2.33, *p* < 0.001). However, the multivariate model showed that age‐related comorbidities were not significant factors for virological failure (hazard ratio 1.31, 95% CI 0.98 to 1.74, *p* = 0.07). There were 391 immunological failures, with an incidence of 2.75/100 person‐years. On multivariate analysis, those aged <50 years without comorbidities (*p* = 0.025) and age <50 years with comorbidities (*p* = 0.001) were less likely to develop immunological failure compared to those aged ≥50 years with comorbidities.

**Conclusions:**

In our Asia regional cohort, age‐associated comorbidities did not affect virologic outcomes of cART. Among those with comorbidities, patients <50 years old showed a better CD4 response.

## 1 Introduction

Combination antiretroviral therapy (cART) has dramatically improved the survival and quality of life for people living with HIV 1, 2, 3. A growing proportion of patients are over the age of 50 years, and by the end of 2013, over four million individuals older than 50 years were living with HIV infection worldwide 4. For instance, in Canada the number of older adults with HIV has doubled over the past 20 years, and in Western Europe the estimated number of people living with HIV aged 50 years and over has almost quadrupled over the past decade 5, 6. Despite successful cART, many ageing HIV‐positive patients have developed age‐associated comorbidities such as cardiovascular, metabolic, pulmonary, renal, bone and malignant diseases, and these are often more prevalent compared with HIV‐negative individuals 7, 8. Risk and management of comorbidities in ageing adults with HIV will continue to evolve as treatment improves and life expectancy increases 5, 6.

Polypharmacy is also common in the HIV‐positive older adult population 9, 10. The Swiss HIV cohort study comparing HIV‐positive adults aged ≥50 years with HIV‐positive patients aged <50 years on cART found that older patients were more likely to receive one or more co‐medications compared with younger patients 11. This study also determined that older patients had more frequent potential for drug‐to‐drug interactions when compared to younger patients. The effects of polypharmacy may be more substantial in older HIV‐positive persons because of the increased chance of drug‐to‐drug interactions 9, 12. It has been shown that older HIV‐positive patients have better adherence to cART than younger patients 13, 14, and this can increase the likelihood of potential drug interactions. Drug interactions might be associated with a substantial risk for toxicity, decreased efficacy and subsequent emergence of drug resistance.

Another paper with the Swiss HIV cohort study investigated the prevalence of comedications and potential drug‐to‐drug interactions within a large HIV cohort, and their effect on ART efficacy and tolerability 15. They found potential drug‐to‐drug interactions increase with complex ART and comorbidities, but no adverse effect was noted on ART efficacy or tolerability.

Previous studies showed older HIV‐positive individuals have a less robust immune response but, likely due to better adherence, a better virologic response 16, 17, 18. However, multiple comorbidities among HIV‐positive individuals may increase the potential for polypharmacy and older individuals with comorbidities, particularly those with cognitive impairment, may have difficulty in adhering to complex medication regimens 13. However, the effects of age‐associated comorbidities on the treatment outcomes of cART are not well known. In this study, we investigated the effects of age‐associated comorbidities on therapeutic outcomes of cART in HIV‐positive adults in Asian countries.

## 2 Methods

### 2.1 Study design and data collection

We analysed data from the TREAT Asia HIV Observational Database (TAHOD), a prospective, observational cohort study of HIV‐positive adults enrolled from 21 clinical sites, which is a contributing cohort to IeDEA Asia‐Pacific 19. We selected eligible subjects for this analysis among patients who were enrolled in TAHOD from 2003 to 2015. The TAHOD database and methods have been previously described 20. Due to the observational nature of the cohort, viral load (VL) and CD4 testing are not performed on a predefined basis but depend on the site's local practices and the patient's financial circumstances. Institutional review board approvals were obtained at all participating sites, the data management and analysis centre (Kirby Institute, University of New South Wales, Sydney, Australia), and the coordinating centre (TREAT Asia/amfAR, Bangkok, Thailand). Patients provided written informed consent to participate in the TAHOD where required by local institutional review boards.

### 2.2 Definitions

Patients were included in the analysis if they had been on cART for more than 6 months. Our failure outcomes were defined to be in‐line with the World Health Organization (WHO) 2016 guidelines 21 as follows: (i) virological failure was defined as a single VL >1000 copies/mL; (ii) immunological failure was defined as CD4 count falling below 250 cells/μL after a clinical failure, or persistent CD4 levels below 100 cells/μL (two consecutive CD4 counts below 100 cells/μL within six months). We assumed no treatment failure had occurred if there was an absence of VL or CD4 count. We utilized a single VL measurement, rather than a second confirmatory testing, as the median VL testing frequency in our cohort was 1 (interquartile range (IQR) 1 to 2) per patient per year. Patients were included in the virological failure analysis if they had at least one VL measurement available after six months on cART. Immunological failure analysis included patients with pre‐cART CD4 count available and at least one CD4 measurement after six months from cART initiation. Both analyses were censored at four years from cART initiation.

Comorbidities evaluated included hypertension, diabetes, dyslipidaemia and impaired renal function. Hypertension was defined as a diastolic blood pressure ≥90 mmHg and/or systolic blood pressure ≥140 mmHg 22; diabetes was defined as a fasting blood glucose level ≥7.0 mmol/L or 126 mg/dL 23; dyslipidaemia was defined using any one of the following four criteria: total cholesterol ≥240 mg/dL, triglyceride ≥200 mg/dL, high‐density lipoprotein cholesterol <40 mg/dL, low‐density lipoprotein cholesterol ≥160 mg/dL according to National Cholesterol Education Programme ATP‐III guidelines; impaired renal function was defined as an estimated glomerular filtration rate (eGFR) <60 mL/minute by CKD EPI equation 24.

Patients were grouped into four categories according to their age and comorbidities: (i) age <50 years with no comorbidities, (ii) age <50 years with comorbidities, (iii) age ≥50 years without comorbidities, and (iv) age ≥50 years with comorbidities. Age‐associated comorbidity and cART adherence were included as time‐varying variables. Time‐fixed covariates included in the analyses were sex, HIV‐1 exposure risks, baseline CD4 cell count, baseline viral load, cART regimen, prior AIDS‐defining illness, hepatitis co‐infection and smoking history. Ethnicity was reported descriptively but not included in the regression analyses due to the inclusion of site as a stratification variable. Year of cART initiation was not included in the multivariate model selection due to collinearity with cART adherence, as our cohort began collecting adherence data from 2011 onwards. However, we assessed the direction of the hazard ratios (HRs) by adjusting with other significant covariates in the absence of the adherence variable.

### 2.3 Statistical analysis

Cox regression analysis was used to analyse time to first virological and immunological failure, stratified by clinical site. Risk time started six months from cART initiation. Patients who did not fail in either category were censored on the last date of VL testing for the virological failure analysis, and of CD4 testing for immunological failure analysis, all within four years from cART initiation. Sensitivity analyses were performed disaggregating by sex. Data management and statistical analyses were conducted using SAS software version 9.4 (SAS Institute Inc., Cary, NC, USA) and STATA software version 14 (STATA Corp., College Station, TX, USA).

## 3 Results

### 3.1 Patient characteristics

Table 1 shows the baseline characteristics of patients included in both the virological and immunological analyses. In the virological failure analysis, a total of 5411 patients were included from Cambodia, China, Hong Kong SAR, India, Indonesia, Japan, Malaysia, the Philippines, Singapore, South Korea, Taiwan, Thailand and Vietnam. The median age at cART initiation was 35 years (IQR 29 to 41), with 66% being aged <50 years without the presence of co‐morbidities prior to cART initiation. Most patients were male (71%) and the majority were Thai (33%) and Chinese (27%). Heterosexual mode of HIV exposure was predominant (62%) and the median CD4 cell count at cART initiation was 130 cells/μL (IQR 40 to 228). Of the 5411 patients, there were 912 (17%) with virological failure. The median age was slightly lower at 34 years (IQR 29 to 40) and the median CD4 cell count was 97 cells/μL (IQR 25 to 200).

**Table 1:** Patient characteristics

	Virological failure		Immunological failure	
	Total patients included = 5411 (100%)	Total patients with VL failures = 912 (17%)	Total patients included = 5621 (100%)	Total patients with immunological failures = 391 (7%)
Age at cART initiation (years)	Median = 35, IQR (29 to 41)	Median = 34, IQR (29 to 40)	Median = 35, IQR (29 to 41)	Median = 35, IQR (30 to 42)
≤30	1616 (30)	317 (35)	1692 (30)	105 (27)
31 to 40	2302 (43)	388 (43)	2417 (43)	168 (43)
41 to 50	1040 (19)	143 (16)	1065 (19)	71 (18)
>50	453 (8)	64 (7)	447 (80)	47 (12)
Year of cART initiation				
<2003	699 (13)	204 (22)	627 (11)	87 (22)
2003 to 2005	1167 (22)	221 (24)	1186 (21)	113 (29)
2006 to 2009	2077 (38)	279 (31)	2207 (39)	139 (36)
2010 to 2014	1468 (27)	208 (23)	1601 (28)	52 (13)
Pre‐cART age‐related comorbidities				
Age <50 years without comorbidities	3559 (66)	623 (68)	3653 (65)	260 (66)
Age <50 years with comorbidities	1335 (25)	219 (24)	1449 (26)	81 (21)
Age ≥50 years without comorbidities	305 (6)	41 (4)	285 (5)	27 (7)
Age ≥50 years with comorbidities	212 (4)	29 (3)	234 (5)	23 (6)
Sex				
Male	3839 (71)	679 (74)	3886 (69)	316 (81)
Female	1572 (29)	233 (26)	1735 (31)	75 (19)
Ethnicity				
Caucasian	19 (0.4)	2 (0.2)	16 (0.3)	1 (0.3)
Chinese	1448 (27)	312 (34)	1284 (23)	112 (29)
Filipino	211 (4)	24 (3)	241 (4)	8 (2)
Indian	503 (9)	87 (10)	734 (13)	55 (14)
Indonesian	236 (4)	68 (7)	405 (7)	53 (14)
Japanese	222 (4)	11 (1)	68 (1)	2 (10
Khmer	218 (4)	22 (2)	436 (8)	50 (13)
Korean	241 (4)	60 (7)	216 (4)	12 (3)
Malay	96 (2)	29 (3)	80 (1)	8 (2)
Thai	1788 (33)	193 (21)	1613 (29)	59 (15)
Vietnamese	401 (7)	103 (11)	502 (9)	31 (8)
Other	28 (1)	1 (0.1)	26 (0.5)	0 (0)
HIV Exposure				
Heterosexual contact	3355 (62)	514 (56)	3736 (66)	290 (74)
Homosexual contact	1385 (26)	218 (24)	1125 (20)	39 (10)
Injecting drug use	289 (5)	103 (11)	335 (6)	38 (10)
Other/Unknown	382 (7)	77 (8)	425 (8)	24 (6)
Pre‐cART Viral Load (copies/mL)	Median = 98,000, IQR (27,700 to 290,000)	Median = 110,000, IQR (33,739 to 390,000)	Median = 99,180, IQR (27,574 to 290,000)	Median = 150,000, IQR (42,089 to 400,000)
<100,000	1570 (29)	226 (25)	1583 (28)	68 (17)
≥100,000	1529 (28)	263 (29)	1561 (28)	99 (25)
Missing	2312 (43)	423 (46)	2477 (44)	224 (57)
Pre‐cART CD4 (cells/μL)	Median = 130, IQR (40 to 228)	Median = 97, IQR (28 to 200)	Median = 127, IQR (40 to 223)	Median = 30, IQR (12 to 73)
≤50	1329 (25)	266 (29)	1642 (29)	254 (65)
51 to 100	636 (12)	108 (12)	801 (14)	56 (14)
101 to 200	1143 (21)	177 (19)	1456 (26)	53 (14)
>200	1463 (27)	182 (20)	1722 (31)	28 (7)
Missing	840 (16)	179 (20)	0	0
Initial cART category				
NRTI+NNRTI	4389 (81)	712 (78)	4845 (86)	343 (88)
NRTI+PI	930 (17)	182 (20)	701 (12)	45 (12)
Other combination	92 (2)	18 (2)	75 (1)	3 (1)
Hepatitis B co‐infection				
Negative	3897 (72)	622 (68)	3903 (69)	287 (73)
Positive	457 (8)	76 (8)	463 (8)	38 (10)
Not tested	1057 (20)	214 (23)	1255 (22)	66 (17)
Hepatitis C co‐infection				
Negative	3635 (67)	555 (61)	3599 (64)	272 (70)
Positive	513 (9)	126 (14)	532 (9)	49 (13)
Not tested	1263 (23)	231 (25)	1490 (27)	70 (18)
Prior AIDS diagnosis				
No	3478 (64)	534 (59)	3571 (64)	177 (45)
Yes	1933 (36)	378 (41)	2050 (36)	214 (55)
Ever smoked cigarettes				
No	2138 (40)	302 (33)	2103 (37)	106 (27)
Yes	1535 (28)	275 (30)	1454 (26)	109 (28)
Unknown	1738 (32)	335 (37)	2064 (37)	176 (45)
cART adherence				
Always ≥95%	2357 (44)	211 (23)	2598 (46)	60 (15)
Ever <95%	253 (5)	53 (6)	270 (5)	16 (4)
Not reported	2801 (52)	648 (71)	2753 (49)	315 (81)

cART, combination antiretroviral therapy; IQR, interquartile range; VL, viral load.

The immunological failure analysis included 5621 patients in total, with a similar distribution of characteristics to the virological failure analysis. The median CD4 testing was two per patient per year (IQR 1 to 2). There were 391 patients (7%) who had an immunological failure. For each of the comorbidity groups, the median CD4 cell count at cART initiation was 116 cells/μL IQR (40 to 209) for age <50 without comorbidities, 157 cells/μL IQR (47 to 245) for age <50 with comorbidities, 116 cells/μL IQR (37 to 217) for age ≥50 years without comorbidities, and 164 cells/μL IQR (61 to 239) for age ≥50 years with comorbidities. Of the total patients in each comorbidity group, the number of patients initiating cART at CD4 cell count ≤200 cells/μL were 2662/3653 (73%), 894/1449 (62%), 198/285 (69%) and 145/234 (62%) respectively.

### 3.2 Virological failure

Of the 5411 patients included, 1858 (34%) had hypertension, 570 (11%) had diabetes mellitus, 2689 (50%) had dyslipidaemia and 353 (7%) had impaired renal function. There were 912 (17%) virological failures reported during 11,814.84 person‐years of follow‐up, with an incidence rate of 7.72 per 100 person‐years (/100PYS) (Table 2). The median time from cART initiation up to date of first virological failure or date of last VL test was three years (IQR 1.7 to 3.7). In the univariate analyses, having age‐related comorbidities (*p* < 0.001), cART adherence (*p* < 0.001), mode of HIV exposure (*p* < 0.001), pre‐ART VL (*p* = 0.029), pre‐ART CD4 (*p* < 0.001), initial ART regimen (*p* = 0.097), hepatitis C co‐infection (*p* = 0.055), prior AIDS diagnosis (*p* = 0.018) and ever smoked (*p* = 0.051) were associated with virological failure, and were thus entered into the multivariate model. Those with adherence <95% (HR = 0.15, 95% confidence interval (CI) 0.10 to 0.21, *p* < 0.001) compared to adherence ≥95% and a higher CD4 count at start of cART (CD4 101 to 200 cells/μL, HR = 0.70, 95% CI 0.57 to 0.85; CD4 > 200 cells/μL, HR = 0.61, 95% CI 0.50 to 0.74, *p* < 0.001) were less likely to have virological failure. Those who acquired HIV through intravenous drug use were more likely to fail compared to a heterosexual mode of exposure (HR = 1.47, 95% CI 1.14 to 1.88, *p* = 0.003). Although not statistically significant, patients aged ≥50 years with comorbidities performed slightly better than the other three groups.

**Table 2:** Factors associated with virological failure

	No patients	Follow‐up (years)	No of failures	Failure rate (per 100 person‐years)	Univariate			Multivariate		
					HR	95% CI	*p*‐value	HR	95% CI	*p*‐value
Total	**5411**	11,814.84	912	7.72						
Age‐related comorbidities							<0.001			0.089
Age <50 years without comorbidities	~	4420.08	441	9.98	1.75	(1.31, 2.33)	<0.001	1.31	(0.98, 1.74)	0.070
Age <50 years with comorbidities	~	5883.95	381	6.48	1.19	(0.90, 1.58)	0.216	1.10	(0.83, 1.45)	0.514
Age ≥50 years without comorbidities	~	375.20	32	8.53	1.54	(0.99, 2.40)	0.054	1.11	(0.71, 1.73)	0.645
Age ≥50 years with comorbidities	~	1135.40	58	5.11	1			1		
cART adherence										
<95%	~	175.01	42	24.00	1			1		
≥95%	~	5923.81	267	4.51	0.14	(0.10, 0.20)	<0.001	**0.15**	**(0.10, 0.21)**	**<0.001**
Missing	~	5716.03	603	10.55						
Sex										
Male	3839	8291.98	679	8.19	1			1		
Female	1572	3522.86	233	6.61	0.97	(0.83, 1.14)	0.727	1.04	(0.87, 1.23)	0.686
HIV exposure							<0.001			**0.019**
Heterosexual contact	3355	7614.28	514	6.75	1			1		
Homosexual contact	1385	3016.02	218	7.23	0.80	(0.65, 0.99)	0.041	1.01	(0.81, 1.25)	0.931
Injecting drug use	289	494.11	103	20.85	1.57	(1.22, 2.01)	<0.001	**1.47**	**(1.14, 1.88)**	**0.003**
Other/Unknown	382	690.43	77	11.15	1.08	(0.83, 1.42)	0.550	1.19	(0.90, 1.56)	0.217
Pre‐cART viral load (copies/μL)										
<100,000	1570	3627.15	226	6.23	1			1		
≥100,000	1529	3369.37	263	7.81	1.22	(1.02, 1.46)	0.029	1.05	(0.87, 1.26)	0.630
Missing	2312	4818.32	423	8.78						
Pre‐cART CD4 (cells/μL)							<0.001			**<0.001**
≤50	1329	2804.11	266	9.49	1			1		
51 to 100	636	1388.94	108	7.78	0.84	(0.67, 1.06)	0.139	0.83	(0.66, 1.04)	0.111
101 to 200	1143	2546.00	177	6.95	0.69	(0.57, 0.84)	<0.001	**0.70**	**(0.57, 0.85)**	**<0.001**
>200	1463	3136.82	182	5.80	0.55	(0.45, 0.67)	<0.001	**0.61**	**(0.50, 0.74)**	**<0.001**
Missing	840	1938.96	179	9.23						
Initial cART category							0.097			0.667
NRTI+NNRTI	4389	9408.65	712	7.57	1			1		
NRTI+PI	930	2193.97	182	8.30	1.24	(1.00, 1.52)	0.048	0.98	(0.79, 1.21)	0.839
Other combination	92	212.22	18	8.48	1.34	(0.83, 2.18)	0.230	1.23	(0.76, 1.99)	0.409
Hepatitis B co‐infection										
Negative	3897	8672.08	622	7.17	1			1		
Positive	457	1023.49	76	7.43	0.98	(0.77, 1.24)	0.850	0.89	(0.70, 1.14)	0.355
Not tested	1057	2119.27	214	10.10						
Hepatitis C co‐infection										
Negative	3635	8261.66	555	6.72	1			1		
Positive	513	980.51	126	12.85	1.24	(1.00, 1.54)	0.055	0.99	(0.77, 1.25)	0.908
Not tested	1263	2572.67	231	8.98						
Prior AIDS diagnosis										
No	3478	7593.57	534	7.03	1			1		
Yes	1933	4221.28	378	8.95	1.18	(1.03, 1.36)	0.018	0.95	(0.81, 1.10)	0.490
Ever smoked cigarettes										
No	2138	4891.63	302	6.17	1			1		
Yes	1535	3466.78	275	7.93	1.18	(1.00, 1.40)	0.051	1.05	(0.88, 1.24)	0.597
Unknown	1738	3456.43	335	9.69						

~ age‐related comorbidity and cART adherence are time‐updated variables. Missing values were included in the regression analyses; however, global *p*‐values were tested for heterogeneity excluding missing categories. Significant *p*‐values are highlighted in bold. Variables not associated with significant *p*‐values are presented in the final table adjusted for the variables with significant *p*‐values. CI, confidence interval; HR, Hazard ratio; NRTI, Nucleoside reverse transcriptase inhibitor; NNRTI, Non‐nucleoside reverse‐transcriptase inhibitor; PI, Protease inhibitor.

To determine the effects of the year of cART initiation on virological failure and to avoid collinearity with the adherence variable, we included year of cART initiation in the multivariate model without adjusting for adherence. As expected, later years of cART initiation were associated with decreased hazard for failure (2003 to 2005: HR = 0.82, 95% CI 0.67 to 1.00, *p* = 0.052; 2006 to 2009: HR = 0.50, 95% CI 0.41 to 0.62, *p* < 0.001; and 2010 to 2014: HR = 0.38, 95% CI 0.29 to 0.49, *p* < 0.001) compared to years prior to 2003.

### 3.3 Immunological failure

The rate of immunological failure was 2.75/100 PYS (Table 3). Of the 5621 patients included, there were 391 (7%) patients who experienced immunological failure during 14,196 person‐years of follow‐up. The median time from cART initiation was 3.5 years (IQR 2.5 to 3.8). There were 2105 (37%) patients with hypertension, 607 (11%) with diabetes, 2748 (49%) with dyslipidaemia and 404 (7%) with impaired renal function.

**Table 3:** Factors associated with immunological failure

	No patients	Follow‐up (years)	No of failures	Failure rate (per 100 person‐years)	Univariate			Multivariate		
					HR	95% CI	*p*‐value	HR	95% CI	*p*‐value
Total	**5621**	**14,196**	**391**	**2.75**						
Age‐related comorbidities							0.003			**0.005**
Age <50 years without comorbidities	~	5310	185	3.48	0.88	(0.62, 1.25)	0.480	**0.66**	**(0.46, 0.95)**	**0.025**
Age <50 years with comorbidities	~	7243	152	2.10	0.62	(0.43, 0.88)	0.007	**0.54**	**(0.38, 0.76)**	**0.001**
Age ≥50 years without comorbidities	~	365	13	3.56	1.01	(0.54, 1.91)	0.968	0.74	(0.39, 1.40)	0.354
Age ≥50 years with comorbidities	~	1279	41	3.21	1			1		
cART adherence										
<95%	~	222	14	6.30	1			1		
≥95%	~	7232	80	1.11	0.15	(0.09, 0.28)	<0.001	**0.16**	**(0.09, 0.29)**	**<0.001**
Missing	~	6742	297	4.41						
Sex										
Male	3886	9644	316	3.28	1			1		
Female	1735	4552	75	1.65	0.51	(0.40, 0.67)	<0.001	**0.60**	**(0.46, 0.79)**	**<0.001**
HIV exposure							<0.001			**0.022**
Heterosexual contact	3736	9654	290	3.00	1			1		
Homosexual contact	1125	2764	39	1.41	0.36	(0.24, 0.55)	<0.001	**0.52**	**(0.34, 0.79)**	**0.002**
Injecting drug use	335	761	38	5.00	1.12	(0.75, 1.67)	0.570	0.86	(0.57, 1.31)	0.490
Other/Unknown	425	1017	24	2.36	0.69	(0.44, 1.08)	0.106	0.76	(0.48, 1.21)	0.248
Pre‐cART viral load (copies/mL)										
<100,000	1583	4023	68	1.69	1			1		
≥100,000	1561	3944	99	2.51	1.37	(1.00, 1.87)	0.051	0.84	(0.61, 1.16)	0.293
Missing	2477	6229	224	3.60						
Pre‐cART CD4 (cells/μL)							<0.001			**<0.001**
≤50	1642	3974	254	6.39	1			1		
51 to 100	801	2046	56	2.74	0.44	(0.33, 0.59)	<0.001	**0.45**	**(0.34, 0.60)**	**<0.001**
101 to 200	1456	3810	53	1.39	0.21	(0.16, 0.29)	<0.001	**0.24**	**(0.17, 0.32)**	**<0.001**
>200	1722	4366	28	0.64	0.09	(0.06, 0.14)	<0.001	**0.11**	**(0.07, 0.17)**	**<0.001**
Initial cART category							0.614			0.923
NRTI+NNRTI	4845	12,163	343	2.82	1			1		
NRTI+PI	701	1851	45	2.43	1.20	(0.82, 1.77)	0.343	0.93	(0.63, 1.36)	0.708
Other combination	75	183	3	1.64	0.91	(0.28, 2.90)	0.872	0.89	(0.28, 2.86)	0.845
Hepatitis B co‐infection										
Negative	3903	10,022	287	2.86	1			1		
Positive	463	1209	38	3.14	1.20	(0.86, 1.69)	0.286	1.01	(0.71, 1.42)	0.977
Not tested	1255	2965	66	2.23						
Hepatitis C co‐infection										
Negative	3599	9324	272	2.92	1			1		
Positive	532	1306	49	3.75	1.11	(0.79, 1.55)	0.555	0.90	(0.61, 1.34)	0.613
Not tested	1490	3565	70	1.96						
Prior AIDS diagnosis										
No	3571	9083	177	1.95	1			1		
Yes	2050	5113	214	4.19	1.78	(1.44, 2.19)	<0.001	0.85	(0.68, 1.07)	0.164
Ever smoked cigarettes										
No	2103	5531	106	1.92	1			1		
Yes	1454	3845	109	2.84	1.23	(0.93, 1.61)	0.142	0.87	(0.65, 1.17)	0.366
Unknown	2064	4820	176	3.65						

~ age‐related comorbidity and cART adherence are time‐updated variables. Missing values were included in the regression analyses, however global *p*‐values were tested for heterogeneity excluding missing categories. Significant *p*‐values are highlighted in bold. Variables not associated with significant *p*‐values are presented in the final table adjusted for the variables with significant *p*‐values. CI, confidence interval; HR, hazard ratio; NRTI, Nucleoside reverse transcriptase inhibitor; NNRTI, Non‐nucleoside reverse‐transcriptase inhibitor; PI, Protease inhibitor.

In the multivariate analyses, those aged <50 years without comorbidities (HR = 0.66, 95% CI 0.46 to 0.95, *p* = 0.025) and aged <50 years with comorbidities (HR = 0.54, 95% CI 0.38 to 0.76, *p* = 0.001) were less likely to develop immunological failure compared to those patients aged ≥50 years with comorbidities. Other factors associated with a reduction in hazard for failure were cART adherence ≥95% (HR = 0.16, 95% CI 0.09 to 0.29, *p* < 0.001) compared to adherence <95%, female sex (HR = 0.60, 95% CI 0.46 to 0.79, *p* < 0.001), homosexual mode of exposure (HR = 0.52, 95% CI 0.34 to 0.79, *p* = 0.002) compared to heterosexual mode of exposure, and higher CD4 count (CD4 51 to 100 cells/μL: HR = 0.45, 95% CI 0.34 to 0.60; CD4 101 to 200 cells/μL: HR = 0.24, 95% CI 0.17 to 0.32; and CD4 > 200 cells/μL: HR = 0.11, 95% CI 0.07 to 0.16, all *p* < 0.001) compared to CD4 ≤ 50 cells/μL.

When year of cART initiation was included in the final multivariate model in place of the adherence variable, we saw decreasing hazard for failure in later years (2003 to 2005: HR = 0.74, 95% CI 0.54 to 1.01, *p* = 0.058; 2006 to 2009: HR = 0.56, 95% CI 0.41 to 0.77, *p* < 0.001; and 2010 to 2014: HR = 0.28 95% CI 0.18 to 0.44, *p* < 0.001) compared to years prior to 2003.

**Figure 1:**
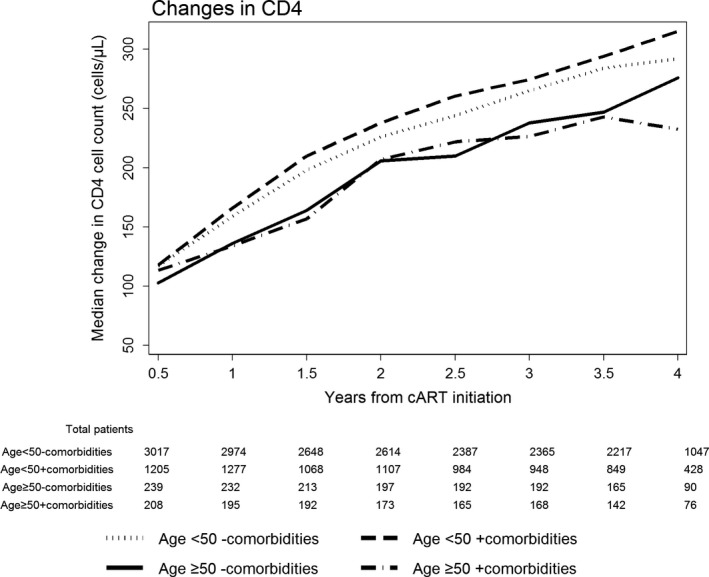
Median changes in CD4 cell count from cART initiation

To examine patterns of CD4 changes in our patient group, we plotted the median change in CD4 cell count for each of our comorbidities group. Figure 1 shows median CD4 increases at each six‐month interval, categorized by comorbidity and age at cART initiation. Patients aged ≥50 years were shown to have slower increases in CD4 cell counts compared to patients <50 years. At four years from cART initiation, patients aged <50 years with comorbidities showed the biggest median change in CD4 cell count, while the median change in the CD4 cell count was the smallest for patients aged ≥50 years with comorbidities. The small decrease in CD4 count in the age ≥50 years with the comorbidities group at the fourth year could be attributed to the small sample size present at that time point (76 patients).

### 3.4 Sensitivity analyses

Factors associated with virological failure in males and females are shown in Table S1. cART adherence <95% was associated with failure in both sexes; however, higher CD4 cell count at cART initiation was associated with reduced hazard for failure in males, but this was not statistically significant in females. Table S2 reports risk factors for immunological failure in males and females. Age <50 with or without comorbidities, cART adherence ≥95%, and higher pre‐cART CD4 cell count were associated with reductions in HRs in both males and females. Females who have never smoked were less likely to develop immunological failure, however this association was not evident in males. Overall the effects of the age‐related comorbidity variable in the main analyses and in the sensitivity analyses remained similar suggesting that regardless of sex, those aged ≥50 years with comorbidities had worse immunological outcomes than their younger counterpart either with or without comorbidities.

## 4 Discussion

We hypothesized that age‐associated comorbidities may worsen therapeutic outcomes of cART, because of the risk of polypharmacy and additive negative effects of these health conditions. However, our results showed that presence of age‐associated comorbidities did not affect virological outcomes of cART, and patients <50 years with comorbidities had better immunological outcomes compared with patients ≥50 years with comorbidities.

The prevalence of age‐related comorbidities in this study population was similar to the results from other studies. The prevalence of dyslipidaemia among HIV‐positive populations differs depending on the methodology and patient population studied, ranging from 20% to 80% 25. According to the Swiss HIV Cohort study, the prevalence of hypertension and diabetes mellitus were 56.3% and 4.1% respectively 1. In that study, the eGFR (calculated by the Modification of Diet in Renal Disease Study equation) of older HIV‐positive participants was lower than that of younger HIV‐positive patients. HIV‐positive patients may have greater risk of non‐infectious comorbidities than the general population, because of the effects of HIV itself, prevalent risk factors, and antiretroviral medications 26. The treatment of older HIV‐positive patients is complicated by pre‐existing comorbid conditions, including cardiovascular, hepatic and metabolic complications that may be exacerbated by the effects of HIV infection per se, immunodeficiency and metabolic and other adverse effects of combination antiretroviral therapy 27, 28. Synergistic deleterious effects of chronic immune activation on the course of HIV infection with the immune senescence of ageing may promote this accelerated course 27.

A study from Italy showed that age‐related non‐infectious comorbidities were more common among HIV‐positive patients than in the general population 26. They performed a case–control study involving ART‐experienced HIV‐positive patients treated from 2002 through to 2009. These patients were compared with age‐, sex‐ and race‐matched adult controls from the general population. The prevalence of hypertension, renal failure and diabetes mellitus of the HIV group <50 years were 13.2%, 3.78% and 6.17% respectively. The rates were greater than the general population.

Multiple studies have demonstrated that, despite successful ART and viral suppression, immune recovery is less robust with increasing age, highlighting the importance of early diagnosis and treatment of HIV 16, 29, 30, 31, 32. Consistent with previous studies, patients aged ≥50 years with comorbidities in our study had a greater rate of immunological failure compared to patients <50 years with comorbidities. As shown in Figure 1, patients aged ≥50 years were shown to have slower increases in CD4 cell counts compared to patients <50 years. This is consistent with previous studies as well. The poorer immune recovery in older populations could be caused in part by decreased thymic function in these groups 31. In addition, late diagnosis can be more frequent in older populations, and low baseline CD4 cells might affects the immunological responses. However, in our study cohort, the highest proportion of those who initiated ART late was in the age <50 years without co‐morbidities group, and the median CD4 cell count at baseline was lowest for those age <50 years without co‐morbidities and those age ≥50 years without co‐morbidities. Nevertheless, older patients derive substantial benefit from cART despite having a less robust immunological response than expected given their adherence to therapy and excellent virological responses 27. cART provides substantial benefit for older and younger HIV‐positive patients 33, and older patients are more likely to achieve virological control of HIV replication 34, 35 and less likely to develop subsequent virological breakthrough 34, findings that correlate with better adherence to therapy by older patients 35. Consistent with previous studies, our study showed that older patients had similar virological outcomes compared with younger patients.

Overall, the effects of the age‐related comorbidity variables in the main analyses and in the sensitivity analyses remained similar, suggesting that regardless of sex, those aged ≥50 years with comorbidities had worse immunological outcomes than their younger counterparts either with or without comorbidities.

The limitations of the study included the presence missing data. As TAHOD is an observational cohort, data collection depends entirely on the standard of care at each individual site. Patients with good clinic attendance may have more frequent comorbidity testing which may lead to earlier or more frequent diagnosis of a comorbidity. Patients with poor clinic attendance may also have these comorbidities present but not detected. As the cohort does not impose specific study procedures or treatment interventions, the study results should be interpreted with this in mind. The cutoff points for virologic and immunologic failures may not necessarily be relevant for individual patient management, but the failure definition is in line with current WHO guidelines for general clinical practice. In addition, the comorbidity variable was defined according to the availability of our data. We were not able to assess the effects of other comorbidities, as we were limited to the data variables being captured in our cohort. Furthermore, our cohort sites are generally urban referral centres. Patients are selected for enrolment based on the likelihood of remaining in care. Therefore, the generalizability of the reported findings is limited.

## 5 Conclusions

Age associated comorbidities did not affect virological outcomes of cART, and older patients with comorbidities were more likely to experience immunological failure compared to those aged <50 years.

## Competing interests

The authors do not have any competing interest to declare.

## Authors’ contributions

MYA, AJ and JYC contributed to the concept development. SK, VK, TTP, RC, AA, NK, WWW, SK, SP, KVN, MPL, AK, FJ, RD, TPM, EY, OTN, BLHS, JT, WR and JYC contributed data for the analysis. AJ performed the statistical analysis. MYA wrote the first draft of the manuscript. All authors commented on the draft manuscript and approved of the final manuscript.

## TAHOD Study members

PS Ly* and V Khol, National Centre for HIV/AIDS, Dermatology & STDs, Phnom Penh, Cambodia; FJ Zhang* †, HX Zhao and N Han, Beijing Ditan Hospital, Capital Medical University, Beijing, China; MP Lee*, PCK Li, W Lam and YT Chan, Queen Elizabeth Hospital, Hong Kong SAR; N Kumarasamy*, S Saghayam and C Ezhilarasi, Chennai Antiviral Research and Treatment Clinical Research Site (CART CRS), YRGCARE Medical Centre, VHS, Chennai, India; S Pujari*, K Joshi, S Gaikwad and A Chitalikar, Institute of Infectious Diseases, Pune, India; S Sangle*, V Mave and I Marbaniang, BJ Government Medical College and Sassoon General Hospital, Pune, India; TP Merati*, DN Wirawan and F Yuliana, Faculty of Medicine Udayana University & Sanglah Hospital, Bali, Indonesia; E Yunihastuti*, D Imran and A Widhani, Faculty of Medicine Universitas Indonesia ‐ Dr. Cipto Mangunkusumo General Hospital, Jakarta, Indonesia; J Tanuma*, S Oka and T Nishijima, National Center for Global Health and Medicine, Tokyo, Japan; JY Choi*, Na S and JM Kim, Division of Infectious Diseases, Department of Internal Medicine, Yonsei University College of Medicine, Seoul, South Korea; BLH Sim*, YM Gani, and R David, Hospital Sungai Buloh, Sungai Buloh, Malaysia; A Kamarulzaman*, SF Syed Omar, S Ponnampalavanar and I Azwa, University Malaya Medical Centre, Kuala Lumpur, Malaysia; R Ditangco*, MK Pasayan and ML Mationg, Research Institute for Tropical Medicine, Muntinlupa City, Philippines; WW Wong*, WW Ku and PC Wu, Taipei Veterans General Hospital, Taipei, Taiwan; OT Ng* ‡, PL Lim, LS Lee and Z Ferdous, Tan Tock Seng Hospital, Singapore; A Avihingsanon*, S Gatechompol, P Phanuphak and C Phadungphon, HIV‐NAT/Thai Red Cross AIDS Research Centre, Bangkok, Thailand; S Kiertiburanakul*, A Phuphuakrat, L Chumla and N Sanmeema, Faculty of Medicine Ramathibodi Hospital, Mahidol University, Bangkok, Thailand; R Chaiwarith*, T Sirisanthana, W Kotarathititum and J Praparattanapan, Research Institute for Health Sciences, Chiang Mai, Thailand; S Khusuwan*, P Kantipong and P Kambua, Chiangrai Prachanukroh Hospital, Chiang Rai, Thailand; W Ratanasuwan* and R Sriondee, Faculty of Medicine, Siriraj Hospital, Mahidol University, Bangkok, Thailand; KV Nguyen*, HV Bui, DTH Nguyen and DT Nguyen, National Hospital for Tropical Diseases, Hanoi, Vietnam; CD Do*, AV Ngo and LT Nguyen, Bach Mai Hospital, Hanoi, Vietnam; AH Sohn*, JL Ross* and B Petersen, TREAT Asia, amfAR ‐ The Foundation for AIDS Research, Bangkok, Thailand; MG Law*, A Jiamsakul* and D Rupasinghe, The Kirby Institute, UNSW Sydney, NSW, Australia.

* TAHOD Steering Committee member; † Steering Committee Chair; ‡ co‐Chair

## Supporting information


**Table S1.** Multivariate analyses for factors associated with virological failure in males and females
**Table S2.** Multivariate analyses for factors associated with immunological failure in males and females
