# Supplementary material for: The influence of age‐associated comorbidities on responses to combination antiretroviral therapy in older people living with HIV
Source: J Int AIDS Soc. 2019 Feb 25;22(2):e25228. doi: 10.1002/jia2.25228 (PMC6389354; doi:10.1002/jia2.25228)
Supplement: Supplementary file 1 — Table S1. Multivariate analyses for factors associated with virological failure in males and females Table S2. Multivariate analyses for factors associated with immunological failure in males and females [file JIA2-22-e25228-s001.docx]

Supplementary table S1: Multivariate analyses for factors associated with virological failure in males and females

|  | **Males** | | | **Females** | | |
| --- | --- | --- | --- | --- | --- | --- |
|  | **HR** | **95% CI** | **p-value** | **HR** | **95% CI** | **p-value** |
| **Age-related comorbidities** |  |  | 0.084 |  |  | 0.251 |
| Age <50 years without comorbidities | 1.46 | (1.04, 2.05) | 0.027 | 0.98 | (0.56, 1.74) | 0.956 |
| Age <50 years with comorbidities | 1.25 | (0.91, 1.73) | 0.171 | 0.72 | (0.40, 1.28) | 0.262 |
| Age ≥50 years without comorbidities | 1.12 | (0.66, 1.90) | 0.668 | 0.97 | (0.42, 2.23) | 0.938 |
| Age ≥50 years with comorbidities | 1 |  |  | 1 |  |  |
| **cART adherence** |  |  |  |  |  |  |
| <95% | 1 |  |  | 1 |  |  |
| ≥95% | **0.15** | **(0.10, 0.22)** | **<0.001** | **0.12** | **(0.06, 0.26)** | **<0.001** |
| Missing |  |  |  |  |  |  |
| **HIV Exposure** |  |  | 0.078 |  |  |  |
| Heterosexual contact | 1 |  |  | 1 |  |  |
| MSM | 1.06 | (0.84, 1.35) | 0.618 | N/A |  |  |
| Injecting drug use | 1.48 | (1.10, 1.98) | 0.009 | N/A |  |  |
| Other/Unknown | 1.08 | (0.77, 1.51) | 0.649 | 1.34 | (0.83, 2.17) | 0.226 |
| **Pre-cART Viral Load (copies/mL)** |  |  |  |  |  |  |
| <100000 | 1 |  |  | 1 |  |  |
| ≥100000 | 1.07 | (0.87, 1.33) | 0.510 | 1.10 | (0.76, 1.59) | 0.624 |
| Missing |  |  |  |  |  |  |
| **Pre-cART CD4 (cells/uL)** |  |  | **<0.001** |  |  | 0.359 |
| ≤50 | 1 |  |  | 1 |  |  |
| 51-100 | **0.77** | **(0.59, 1.00)** | **0.047** | 1.01 | (0.63, 1.63) | 0.955 |
| 101-200 | **0.69** | **(0.55, 0.87)** | **0.002** | 0.72 | (0.48, 1.07) | 0.107 |
| >200 | **0.54** | **(0.43, 0.68)** | **<0.001** | 0.85 | (0.55, 1.30) | 0.444 |
| Missing |  |  |  |  |  |  |
| **Initial cART Category** |  |  | 0.654 |  |  | 0.086 |
| NRTI+NNRTI | 1 |  |  | 1 |  |  |
| NRTI+PI | 1.06 | (0.84, 1.33) | 0.643 | 0.56 | (0.34, 0.94) | 0.027 |
| Other combination | 1.28 | (0.74, 2.23) | 0.383 | 0.91 | (0.33, 2.56) | 0.862 |
| **Hepatitis B co-infection** |  |  |  |  |  |  |
| Negative | 1 |  |  | 1 |  |  |
| Positive | 0.90 | (0.69, 1.17) | 0.424 | 0.90 | (0.48, 1.68) | 0.745 |
| Not tested |  |  |  |  |  |  |
| **Hepatitis C co-infection** |  |  |  |  |  |  |
| Negative | 1 |  |  | 1 |  |  |
| Positive | 1.09 | (0.84, 1.40) | 0.520 | 1.25 | (0.74, 2.12) | 0.410 |
| Not tested |  |  |  |  |  |  |
| **Prior AIDS Diagnosis** |  |  |  |  |  |  |
| No | 1 |  |  | 1 |  |  |
| Yes | 0.87 | (0.73, 1.04) | 0.128 | 1.16 | (0.87, 1.55) | 0.319 |
| **Ever smoked cigarettes** |  |  |  |  |  |  |
| No | 1 |  |  | 1 |  |  |
| Yes | 1.11 | (0.90, 1.37) | 0.307 | 0.92 | (0.53, 1.58) | 0.760 |
| Unknown |  |  |  |  |  |  |

Note: age-related comorbidity and cART adherence are time-updated variables.

Missing values were included in the regression analyses, however global p-values were test for heterogeneity excluding missing categories.

Significant p-values are highlighted in bold. Variables not associated with significant p-values are presented in the final table adjusted for the variables with significant p-values.

NRTI: Nucleoside reverse transcriptase inhibitor; NNRTI: Non-nucleoside reverse-transcriptase inhibitor; PI: Protease inhibitor

Supplementary table S2: Multivariate analyses for factors associated with immunological failure in males and females

|  | **Males** | | | **Females** | | |
| --- | --- | --- | --- | --- | --- | --- |
|  | **HR** | **95% CI** | **p-value** | **HR** | **95% CI** | **p-value** |
| **Age-related comorbidities** |  |  | **0.058** |  |  | **0.001** |
| Age <50 years without comorbidities | **0.67** | **(0.45, 1.01)** | **0.056** | **0.32** | **(0.12, 0.83)** | **0.019** |
| Age <50 years with comorbidities | **0.59** | **(0.40, 0.86)** | **0.007** | **0.15** | **(0.05, 0.44)** | **0.001** |
| Age ≥50 years without comorbidities | 0.61 | (0.29, 1.29) | 0.196 | 0.84 | (0.21, 3.48) | 0.815 |
| Age ≥50 years with comorbidities | 1 |  |  | 1 |  |  |
| **cART adherence** |  |  |  |  |  |  |
| <95% | 1 |  |  | 1 |  |  |
| ≥95% | **0.22** | **(0.11, 0.43)** | **<0.001** | **0.04** | **(0.01, 0.16)** | **<0.001** |
| Missing |  |  |  |  |  |  |
| **HIV Exposure** |  |  | **0.016** |  |  |  |
| Heterosexual contact | 1 |  |  | 1 |  |  |
| MSM | **0.50** | **(0.32, 0.77)** | **0.002** | N/A |  |  |
| Injecting drug use | 0.96 | (0.61, 1.51) | 0.859 | N/A |  |  |
| Other/Unknown | 0.69 | (0.41, 1.17) | 0.164 | 0.65 | (0.23, 1.85) | 0.420 |
| **Pre-cART Viral Load (copies/mL)** |  |  |  |  |  |  |
| <100000 | 1 |  |  | 1 |  |  |
| ≥100000 | 0.80 | (0.57, 1.13) | 0.209 | 1.28 | (0.53, 3.10) | 0.585 |
| Missing |  |  |  |  |  |  |
| **Pre-cART CD4 (cells/uL)** |  |  | **<0.001** |  |  | **<0.001** |
| ≤50 | 1 |  |  | 1 |  |  |
| 51-100 | **0.45** | **(0.33, 0.63)** | **<0.001** | **0.32** | **(0.16, 0.65)** | **0.001** |
| 101-200 | **0.25** | **(0.18, 0.35)** | **<0.001** | **0.15** | **(0.08, 0.31)** | **<0.001** |
| >200 | **0.11** | **(0.07, 0.17)** | **<0.001** | **0.11** | **(0.05, 0.25)** | **<0.001** |
| **Initial cART Category** |  |  | 0.785 |  |  |  |
| NRTI+NNRTI | 1 |  |  | 1 |  |  |
| NRTI+PI | 0.87 | (0.57, 1.33) | 0.526 | 0.93 | (0.35, 2.46) | 0.888 |
| Other combination | 1.12 | (0.35, 3.63) | 0.847 | N/A |  |  |
| **Hepatitis B co-infection** |  |  |  |  |  |  |
| Negative | 1 |  |  | 1 |  |  |
| Positive | 1.02 | (0.71, 1.47) | 0.904 | 0.68 | (0.21, 2.26) | 0.532 |
| Not tested |  |  |  |  |  |  |
| **Hepatitis C co-infection** |  |  |  |  |  |  |
| Negative | 1 |  |  | 1 |  |  |
| Positive | 0.94 | (0.62, 1.45) | 0.794 | 0.65 | (0.15, 2.79) | 0.562 |
| Not tested |  |  |  |  |  |  |
| **Prior AIDS Diagnosis** |  |  |  |  |  |  |
| No | 1 |  |  | 1 |  |  |
| Yes | 0.87 | (0.68, 1.11) | 0.259 | 0.94 | (0.57, 1.57) | 0.825 |
| **Ever smoked cigarettes** |  |  |  |  |  |  |
| No | 1 |  |  | 1 |  |  |
| Yes | 0.93 | (0.67, 1.27) | 0.640 | **0.10** | **(0.01, 0.79)** | **0.029** |
| Unknown |  |  |  |  |  |  |

Note: age-related comorbidity and cART adherence are time-updated variables.

Missing values were included in the regression analyses, however global p-values were test for heterogeneity excluding missing categories.

Significant p-values are highlighted in bold. Variables not associated with significant p-values are presented in the final table adjusted for the variables with significant p-values.

NRTI: Nucleoside reverse transcriptase inhibitor; NNRTI: Non-nucleoside reverse-transcriptase inhibitor; PI: Protease inhibitor
